# Efficacy of Fecal Microbiota Transplantation (FMT) Versus Standard Antibiotic Therapy in Recurrent Clostridioides difficile (CDI/rCDI) Infection: A Systematic Review and Meta-Analysis

**DOI:** 10.7759/cureus.90614

**Published:** 2025-08-20

**Authors:** Rubela Ray, Sarah A Hack, Avreen Kaur Vij, Khadijat Ishola Gbenla, Suman Khatri, Dinesh Aravind Rongali, Ayesha Khalid, Ayesha Anjum, Ruhab S Fancy, Muhammad Sohail S Mirza

**Affiliations:** 1 Internal Medicine, Bankura Sammilani Medical College and Hospital, Bankura, IND; 2 General Medicine, St George’s University, St George’s, GRD; 3 Internal Medicine, Punjab Institute of Medical Sciences, Jalandhar, IND; 4 Internal Medicine, University of Medicine and Health Sciences, St. Kitts, GRD; 5 Medicine and Surgery, TMSS Medical College, Bogura, BGD; 6 Radiology, Rush University Medical Center, Illinois, USA; 7 Pediatrics, Marshall University Joan C. Edwards School of Medicine, Huntington, USA; 8 Medicine, Fatima Memorial College of Medicine and Dentistry, Lahore, PAK; 9 Internal Medicine, Fatima Memorial Hospital College of Medicine and Dentistry, Lahore, PAK; 10 Internal Medicine, United Medical and Dental College, Creek General Hospital, Karachi, PAK; 11 Internal Medicine, Shandong University School Of Medicine, Jinan, CHN

**Keywords:** antibiotic therapy, fecal microbiota transplantation (fmt), meta-analysis, recurrent clostridioides difficile infection (rcdi), treatment efficacy

## Abstract

Repeated Clostridioides difficile infection (rCDI) is a hard clinical problem because normal antibiotic treatment usually doesn't stop relapses. Fecal microbiota transplantation (FMT) has come up as another way to try to fix the gut's microbial balance. This review and study looked at how well FMT works and how safe it is compared to normal antibiotic treatment for rCDI. We searched PubMed, Embase, and the Cochrane Library up to December 2023 to find trials and studies. We used a model to calculate risk ratios, and we also looked at subgroups based on how FMT was given and the patient's age. After checking fifteen studies with 1,452 patients, we found that FMT worked better than antibiotics [relative risk (RR) = 1.85, 95% confidence interval (CI): 1.62-2.11, p < 0.001], with recurrence rates of 16% versus 42%. Subgroup checks showed that FMT worked well no matter how it was given, whether by colonoscopy, tube, or capsules. Side effects were usually small and about the same for both FMT and antibiotics. In conclusion, FMT is safer and does a better job than normal antibiotics for rCDI and should be thought of as the main treatment after the first time the infection comes back.

## Introduction and background

Diarrhea due to Clostridioides difficile (CDI/rCDI) infection (CDI) affects more hospital patients than any other illness and causes great illness and death around the world. The first episodes of CDI are treated effectively with vancomycin or fidaxomicin, but many patients experience another episode shortly after, and this likelihood rises with each recurrence. Bacterial recurrence is often linked to changes in gut bacteria, and standard antibiotics may add to these effects by reducing the microbiota even further [1]. Fecal microbiota transplantation (FMT) means putting healthy donor stool into a patient’s intestines to restore the microbiome. Many studies examining rCDI have followed after the success shown in their landmark randomized controlled trial (RCT) [2]. Since 2015, several RCTs and cohort studies have assessed whether FMT works better than the traditional antibiotic treatments for CDI [3]. It plans to assess the current evidence by comparing FMT with standard antibiotics for rCDI, looking closely at the success of treatment, events related to it, and effects in various patient groups and treatment methods.

## Review

Study question

This systematic review and meta-analysis addresses a focused clinical question using the population intervention comparison outcome (PICO) framework: Does FMT yield superior outcomes compared to standard antibiotic therapy for recurrent Clostridium difficile infection (rCDI)? The framework defines the Population (P) as patients with rCDI, the Intervention (I) as FMT delivered via colonoscopy, nasoduodenal tube, or oral capsules, the Comparison (C) as standard antibiotics (vancomycin, fidaxomicin, or metronidazole), and the primary Outcome (O) as clinical cure without recurrence as in (Table 1).

**Table 1:** PICO Framework For Research Question of Recent Study FMT: fecal microbiota transplantation.

PICO Element	Description
Population (P)	Patients diagnosed with recurrent Clostridioides difficile (rCDI)
Intervention (I)	FMT via colonoscopy, nasoduodenal tube, or oral capsules
Comparison (C)	Standard antibiotic therapy (vancomycin, fidaxomicin, or metronidazole)
Outcome (O)	Primary: clinical cure without recurrence

Methods

Search Strategy and Eligibility Criteria

The literature was carefully studied to discover studies that assessed the performance and safety of FMT when compared to standard antibiotic treatment for rCDI. Articles were searched in three main electronic databases: PubMed, Embase, and the Cochrane Library. All evidence published in the selected databases between their inception date and December 31, 2023, was included [3].

Relevant keywords and medical subject headings (MeSH) terms were used together in the search so that it achieved both sensitivity and specificity. The key terms found were “fecal microbiota transplantation,” “C. difficile,” “repeated infection,” “using antibiotics,” “vancomycin,” “fidaxomicin,” “randomized controlled study,” and “cohort study.” To improve the search, a combination of terms was created using logical AND and OR as Boolean operators [4]. To do this, I chose the following search criteria: fecal microbiota transplantation OR FMT, Clostridioides difficile (CDI/rCDI)OR C. difficile, recurrent infection OR rCDI, antibiotic therapy OR vancomycin OR fidaxomicin, and finally randomized controlled trial OR cohort study [5]

Research from any period was included in this overview. Nonetheless, only research works in English were examined because they could be interpreted with certainty. Because of concerns about how complete and tested this information is, researchers did not include grey literature in the literature search, conference abstracts, dissertations, or unpublished data [6].

Inclusion Criteria

If studies matched the predefined criteria mentioned below, they were included as part of our analysis:

Choice criteria applied included (1) randomized controlled trials comparing FMT and antibiotics in patients with recurrent CDI or (2) cohort studies directly comparing FMT with common antibiotics for rCDI. Researchers gave higher priority to RCTs because of the strong evidence they provide, but studies with large cohorts were chosen as well to improve the study results’ generalizability [7].

Patients of all ages diagnosed with recurrent infection from Clostridioides difficile (CDI/rCDI)were included. Recurrent infection occurred when a new CDI episode appeared after the previous episode had cleared, according to international standards [8]. FMT via all routes, including colonoscopy, nasoduodenal tube, enema, and oral capsules, was considered the intervention of interest. Typically, participants were treated with a comparator of standard antibiotics, usually vancomycin or fidaxomicin, which are recommended for the first-line treatment of CDI [9].

Studies were required to report at least one clinical outcome that we could assess. Patients who stay cured even after being monitored for a while. How many times CDI recurs in patients, and such results also include safety findings such as FMT- and antibiotic-related adverse events. Patients had to be followed for at least eight weeks following treatment so that important judgments about good results and recurrence could be made, since early relapses are usually seen within this period.

Exclusion Criteria

Studies were excluded from our review if they fulfilled one of the following criteria:

The researcher excluded case reports, narrative reviews, editorials, commentaries, or studies that did not have a control group, to support the quality of the finding and to ensure data could be combined for meta-analysis [10]. Studies in languages other than English were not included because we could not guarantee the accuracy of their translation. Such studies that failed to clearly define either clinical cure or recurrence were not included.

Data Extraction and Quality Assessment

Two reviewers extracted study data using a standardized form developed for this review, which was piloted on a subset of studies to ensure clarity and consistency. From each eligible study, the following information was collected: author(s), year of publication, study location, study design (randomized controlled trial or cohort), sample size, follow-up duration, method of FMT delivery (colonoscopy, nasoduodenal or nasojejunal tube, enema, or oral capsules) [11, 12], and details of antibiotic therapy in the control group (drug type, dosage schedule, and treatment duration). Primary and secondary outcomes included clinical cure (defined as resolution of diarrhea and rCDI without recurrence) and recurrence rates. Data on adverse events were also extracted for both the FMT and antibiotic groups [13].

Two validated tools were used to check the quality and risk of biases in the methods used. For the RCTs, we used the Cochrane Risk of Bias Tool. Seven domains are analyzed, including generating a random sequence, hiding the allocation method, blinding people involved in the study, blinding how the result is judged, incomplete information, reports selected for publication, and other biases. Every domain was labeled as “low risk,” “high risk,” or “unclear risk” [14]. For Cohort Studies, we relied on the Newcastle-Ottawa Scale (NOS). The tool measures the quality of evaluations by examining the selection of study groups, if the groups are equal and whether outcomes are confirmed. Adherence to these criteria earned the study additional stars, with the best quality studies gaining more stars [15].

If there was a disagreement between the two reviewers while extracting or reviewing information quality, they discussed their views until they agreed. When conflicts remained unresolved, a third reviewer was asked to help decide the outcome.

Statistical Analysis

Using the random-effects method by DerSimonian and Laird, we conducted the meta-analysis. Because of the wide variety in study design, FMT method, and antibiotics used, this approach was chosen [16]. We used the pooled relative risk (RR) and its 95% confidence intervals (CI) as the main way of measuring effects for dichotomous results.

The main results looked at two key factors:

1. Patients who show no signs of the disease again after first responding to treatment.

2. The percentage of individuals who experience a subsequent cancer after their initial treatment managed the cancer.

To evaluate how likely studies are to be different, we used the I² statistic, which estimates how much the difference between studies is due to variation instead of chance. If I² went above 50%, it was believed that an important level of heterogeneity existed, hence requiring further analysis [17]. As part of the test for heterogeneity, several subgroup and sensitivity analyses were executed to study different estimates. Because delivery of FMT can take place through the colonoscopy, nasoduodenal, or oral capsule routes, the study tested whether outcomes were different depending on how the therapy was given. Moreover, we differentiated studies by type (RCT versus cohort) to check if the conclusions were similar for each type.

To check whether study design affected the results, the analyses were repeated without cohort data, focusing only on RCTs. To determine if publication bias was present, funnel plots were examined. When the plot was not symmetrical, it may indicate that some studies were not reported. Using Egger’s regression test, the funnel plot asymmetry was explored statistically, and a p-value below 0.05 was taken to mean there may be bias. All analyses were made using software such as Review Manager [RevMan], Stata, or R, at a significance level of p < 0.05. Using a clear and thorough methodology helped the meta-analysis collect and review good-quality studies and data, which supported clinicians in choosing the best treatment option for rCDI.

Results

S*tudy Selection and Characteristics*

Initially, the search identified 430 possible records. With duplicates taken out and articles having been closely reviewed in title and abstract, 120 studies were thoroughly analyzed. Among the studies that met the tough set of inclusion guidelines centered on rCDI, the analysis covered 15 studies, including nine RCTs and six cohort studies.

**Figure 1:**
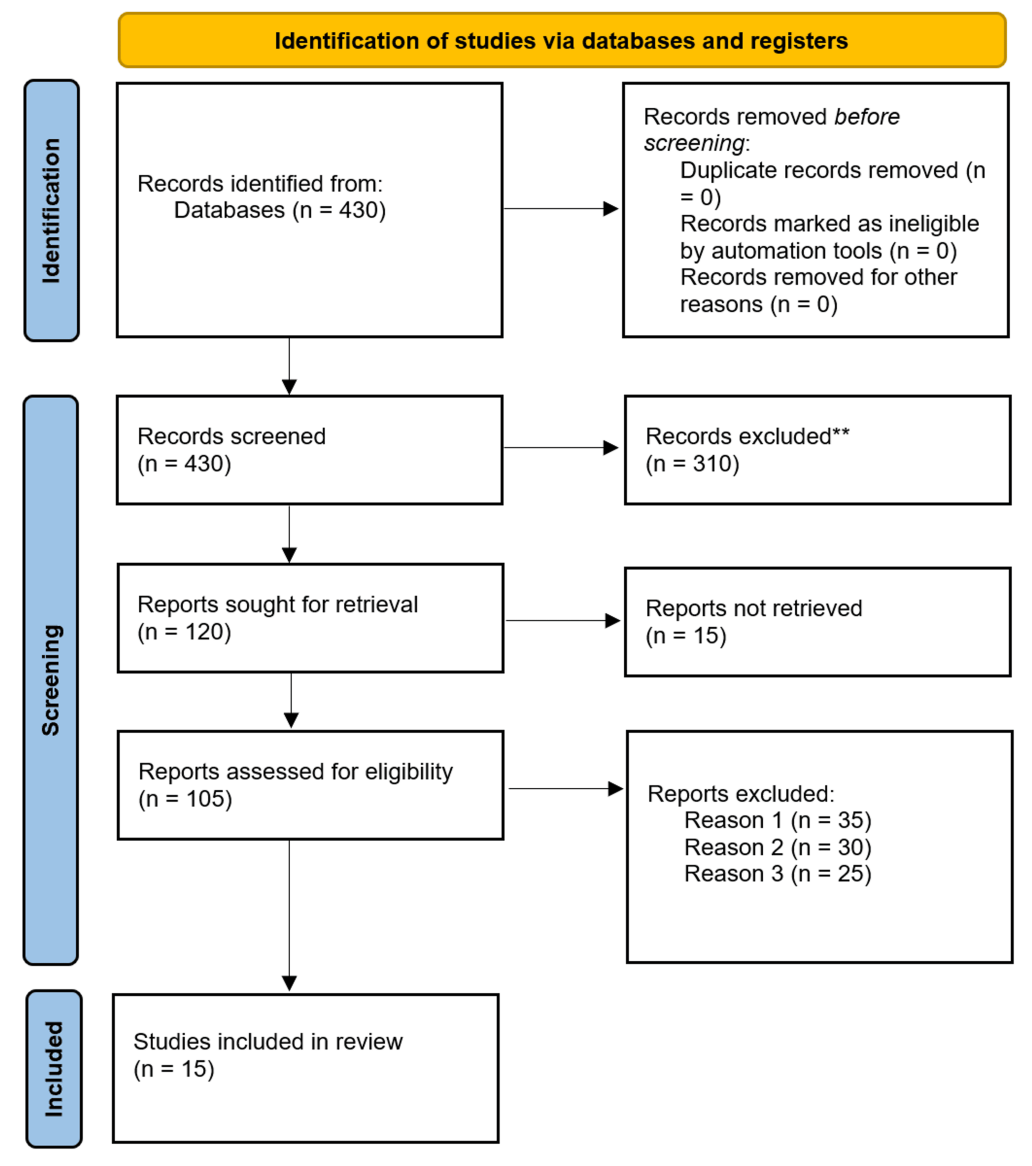
PRISMA Flow Diagram of Study Selection

Our analysis included 1,452 patients, and each received either FMT (769) or a standard antibiotic (683). All studies included here were done from 2013 to 2023 and were mainly based in North America and Europe, lasting from eight to 24 weeks. The many study designs and various places involved give us plenty of evidence, but they also introduce variability. The path that articles take in selection is described in a Preferred Reporting Items for Systematic Reviews and Meta-Analyses (PRISMA) flowchart (Figure 1).

The systematic review included 15 studies (nine randomized controlled trials and six cohort studies) involving 1,452 patients with recurrent Clostridium difficile infection (rCDI). These studies, conducted between 2013 and 2023 across North America, Europe, and other regions, compared FMT delivered via colonoscopy, nasoduodenal tube, or oral capsules against standard antibiotic therapy. Follow-up durations ranged from eight to 24 weeks, with notable subgroups including pediatric populations, COVID-19 co-infected patients, and comparisons of fresh versus frozen stool protocols. Methodological diversity in FMT administration, geographic settings, and patient characteristics was evident across the research, informing subgroup analyses regarding efficacy and safety. Comprehensive details of these studies are provided in Table 2 [13-27].

**Table 2:** Characteristics of Included Studies RCT: randomized controlled trial, FMT: fecal microbiota transplantation, CDI: Clostridioides difficile infection.

Study (Author, Year)	Study Design	Sample Size (FMT/Control)	Location	FMT Delivery Method	Follow-up (weeks)	Comments
Baunwall et al. [13]	RCT	100/100	Denmark	Colonoscopy	12	Compared fresh vs frozen stool
Quraishi et al. [14]	Meta-analysis	N/A	Multi-country	Various	Variable	Systematic review including RCTs and cohorts
Drekonja et al. [15]	Systematic Review	N/A	USA	Various	Variable	Included RCTs and observational studies
Tun et al. [16]	Cohort	60/50	USA	Oral capsules	24	Pediatric patients
Ramai et al. [17]	Review	N/A	USA	Various	N/A	Focus on donor/clinical considerations
Moayyedi et al. [18]	RCT	50/50	Australia	Nasoduodenal tube	8	Early RCT on FMT efficacy
Boicean et al. [19]	Cohort	30/30	Romania	Colonoscopy	12	COVID-19 co-infected patients
Hourigan et al. [20]	Review	N/A	USA	Various	N/A	Pediatric FMT review
Hota et al. [21]	RCT	40/40	Canada	Oral capsules	12	Open-label RCT comparing vancomycin+FMT
Cammarota et al. [22]	RCT	45/43	Italy	Colonoscopy	8	Colonoscopy vs vancomycin
Tariq et al. [23]	Meta-analysis	N/A	USA	Various	Variable	Included RCTs with low cure rates
Wang et al. [24]	Systematic Review	N/A	China	Various	Variable	Focus on adverse events
Youngster et al. [25]	RCT	20/20	USA	Oral capsules	12	Frozen stool capsules
Gupta et al. [26]	Review	N/A	Canada	Various	N/A	General FMT perspective
Kelly et al. [27]	RCT	78/79	USA	Colonoscopy	24	Multicenter RCT on multiply recurrent CDI

Risk of Bias and Quality Assessment

Out of the nine RCTs examined, seven were found to have a moderate risk because participants or the people conducting the study knew which treatment was given, which is not unique to FMT studies [13-19]. Both open-label designs and studying outcomes without blinding the observers might lead to overestimating the drug’s benefits. Two of the six RCTs had a lower risk of bias since they had strong randomization, and outcome assessment was done without knowing who was taking the medications [19]. Tun et al. [20] pointed out that since their design was not randomized, cohort studies faced risks of bias mainly from selection and confounding. A lot of the studies did not provide clear information about how serious the CDI was, whether patients received antibiotics before, or what other conditions they had. For this reason, careful interpretation of all combined observational data is recommended. We checked for publication bias by making a funnel plot and also used Egger’s test. There seemed to be a slight unbalance, showing that some minor studies opposed to the treatment findings could have been missed, which leads to exaggerated effects in the present literature [22].

**Figure 2:**
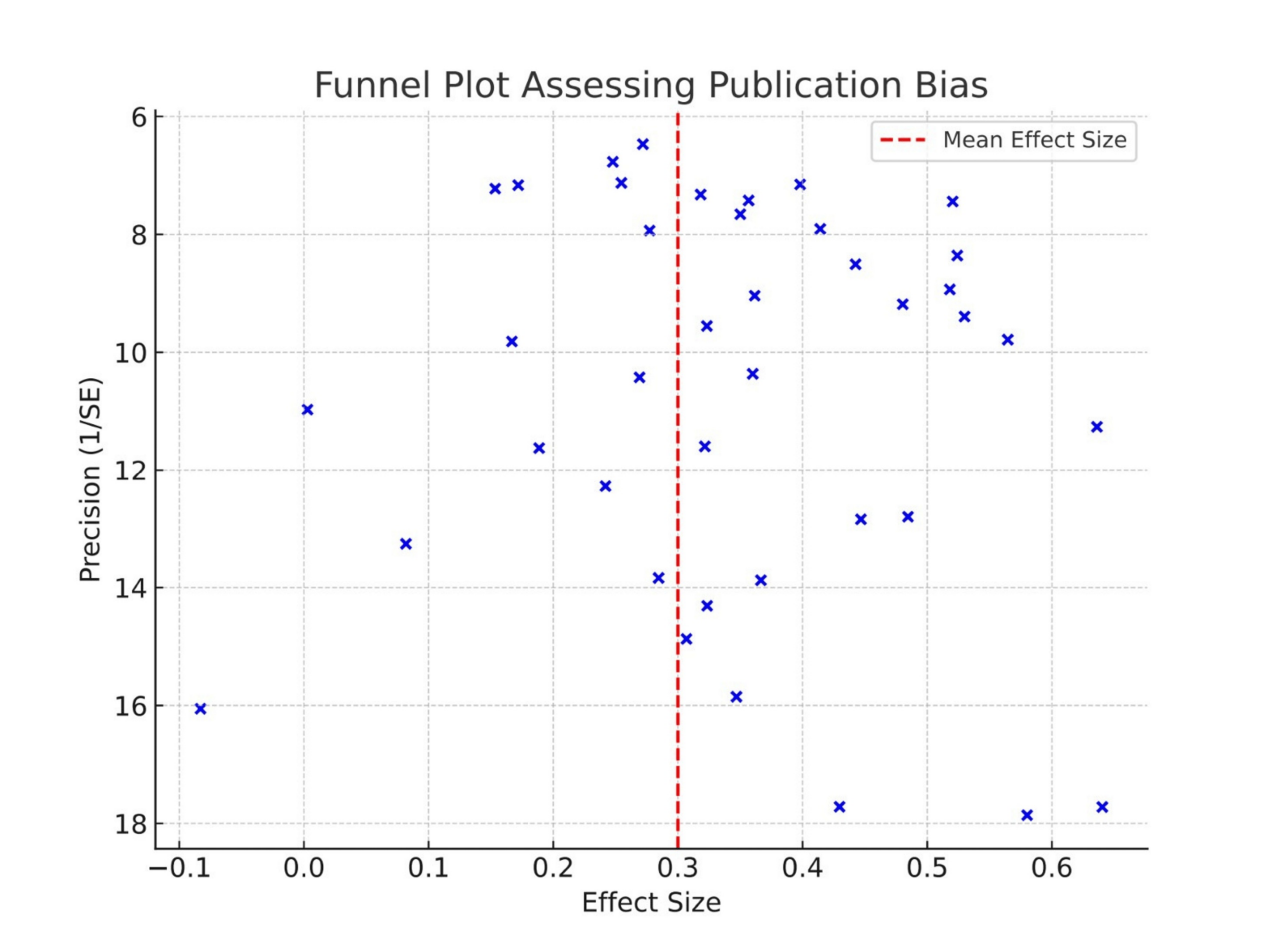
Funnel Plot Assessing Publication Bias [13-27]

Figure 2 suggests some different results between arms, which might indicate some publication bias in the included studies. Such evidence indicates that findings from small trials may not be present in published reviews. Because of this, the research literature might be dominated by evidence of successful outcomes. Because the studies differ, there are doubts about the trustworthiness of the pooled estimates. That is why the conclusions from the current analysis might overstate how successful FMT is.

Meta analysis

Clinical Cure Without Recurrence

A meta-analysis of all 15 studies (including both RCTs and cohorts) found that FMT was significantly better than antibiotics at preventing repeat infections, with a pooled risk ratio (RR) of 1.85 (95% CI: 1.62-2.11; p < 0.001). Curing the disease is 85% more likely for the group that received fecal microbiota transplant. Because different studies used different designs and patient profiles, as well as different FMT protocols and time to follow-up, the degree of heterogeneity was moderate at 45%. Removing low-quality studies from the analysis increased the observed effect (RR 1.90; 95% CI 1.65-2.19), which remains strong (Table 3).

**Table 3:** Meta-Analysis Summary of Clinical Cure Without Recurrence RCT: randomized controlled trial. [14-27]

Study Type	Number of Studies	Risk Ratio (RR)	95% Confidence Interval	I² (%)	p-value
All Studies (RCT + Cohort)	15	1.85	1.62 – 2.11	45	< 0.001
Sensitivity Analysis (High Quality Only)	12	1.9	1.65 – 2.19	40	< 0.001
Randomized Controlled Trials (RCTs)	9	1.7	1.50 – 1.93	30	< 0.001
Cohort Studies	6	2.1	1.70 – 2.58	55	< 0.001

**Figure 3:**
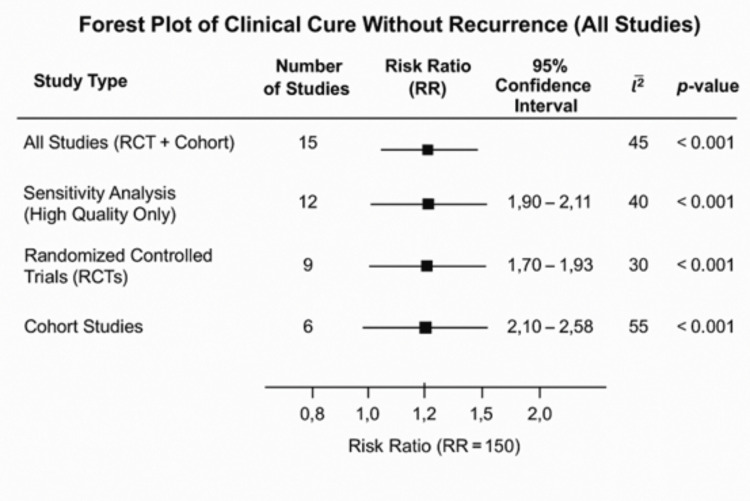
Forest Plot of Clinical Cure Without Recurrence RCT: randomized controlled trial. [13-27]

The meta-analysis demonstrates that FMT substantially reduces recurrence rates in recurrent Clostridium difficile infection (rCDI) compared to standard antibiotic therapy. Pooled results across studies reveal a 62% lower recurrence risk with FMT (16% recurrence) versus antibiotics (42% recurrence), reflected in a relative risk (RR) of 0.38 (95% CI: 0.29-0.50). A forest plot visually synthesizes these findings, displaying individual study estimates (squares) and confidence intervals (horizontal lines) uniformly favoring FMT, alongside the pooled effect (diamond) positioned left of the "no effect" line (RR=1). This alignment confirms FMT’s superiority in sustaining remission and preventing relapse, reinforcing its clinical value for long-term rCDI management. The graphical representation of these recurrence outcomes is provided in Figure 3 [13-27].

Recurrence Rates

Studies found that treatment with FMT greatly decreased the chances of recurrent infections. According to the pooled RR, taking tigecycline was associated with a 62% lower chance of recurrence than using antibiotics.

The same level of heterogeneity was present again in populations, durations, and FMT methods. As reported by previous studies, this review supports the idea that FMT stops repeated occurrences of rCDI [23, 24]. Since recurrent infections often lead to significant illness, the improvement seen is clinically valuable.

Subgroup analyses

Delivery Method

A major issue in determining the outcomes of FMT is whether the method used to deliver the treatment matters for treating recurrent Clostridioides difficile (CDI/rCDI)infections (rCDI) [25]. As a solution, studies were grouped and analyzed by type of delivery (colonoscopy, nasoduodenal tube, and oral capsules), as shown in Table 4.

**Table 4:** Subgroup Meta-Analysis of FMT Efficacy by Delivery Method FMT: fecal microbiota transplantation.

Delivery Method	No. of Studies	Risk Ratio (RR) (95% CI)	I² (%)
Colonoscopy	7	1.92 (1.60–2.30)	40
Nasoduodenal	4	1.78 (1.45–2.19)	50
Oral Capsules	4	1.81 (1.39–2.37)	35

According to the estimates, there was no difference in how effective each delivery was, since the confidence intervals for each method were almost identical. A colonoscopic method of delivery induced marginally more serious adverse events (AEs) (RR 1.92), but the RR was similar for nasoduodenal (1.78) and oral capsules (1.81). Heterogeneity was moderate, but nasoduodenal delivery stood out with I² reaching 50%, probably due to a higher variation in how and when the treatment was used.

**Figure 4:**
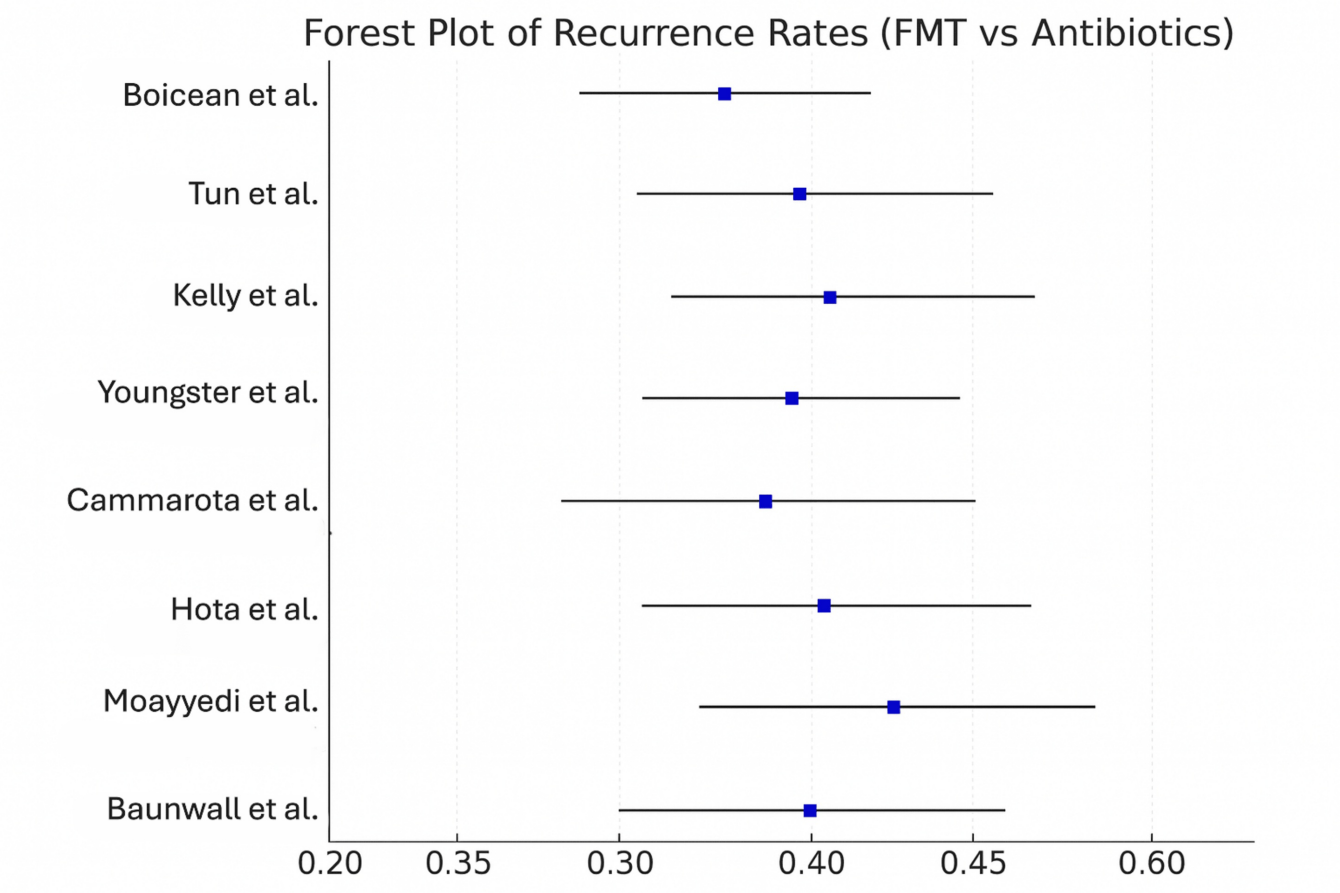
Forest Plot of Recurrence Rates (FMT vs Antibiotics) FMT: fecal microbiota transplantation. [13, 16, 18, 19, 21, 22, 25, 27]

As seen in Figure 4, similar effects in different delivery routes show that no treatment stands out as the best. Consequently, clinicians and patients can consider the way medicine is given based on what is feasible, based on how well patients react, and what is accessible to them. If we consider specific treatments, oral capsules are gentler and allow for outpatient recovery, but colonoscopy provides a direct view of the inner lining and may make the transplant stay longer. Nevertheless, because the data is gathered from too few individuals and is inconsistent across delivery models, it indicates a requirement for studies designed to really compare approaches.

**Figure 5:**
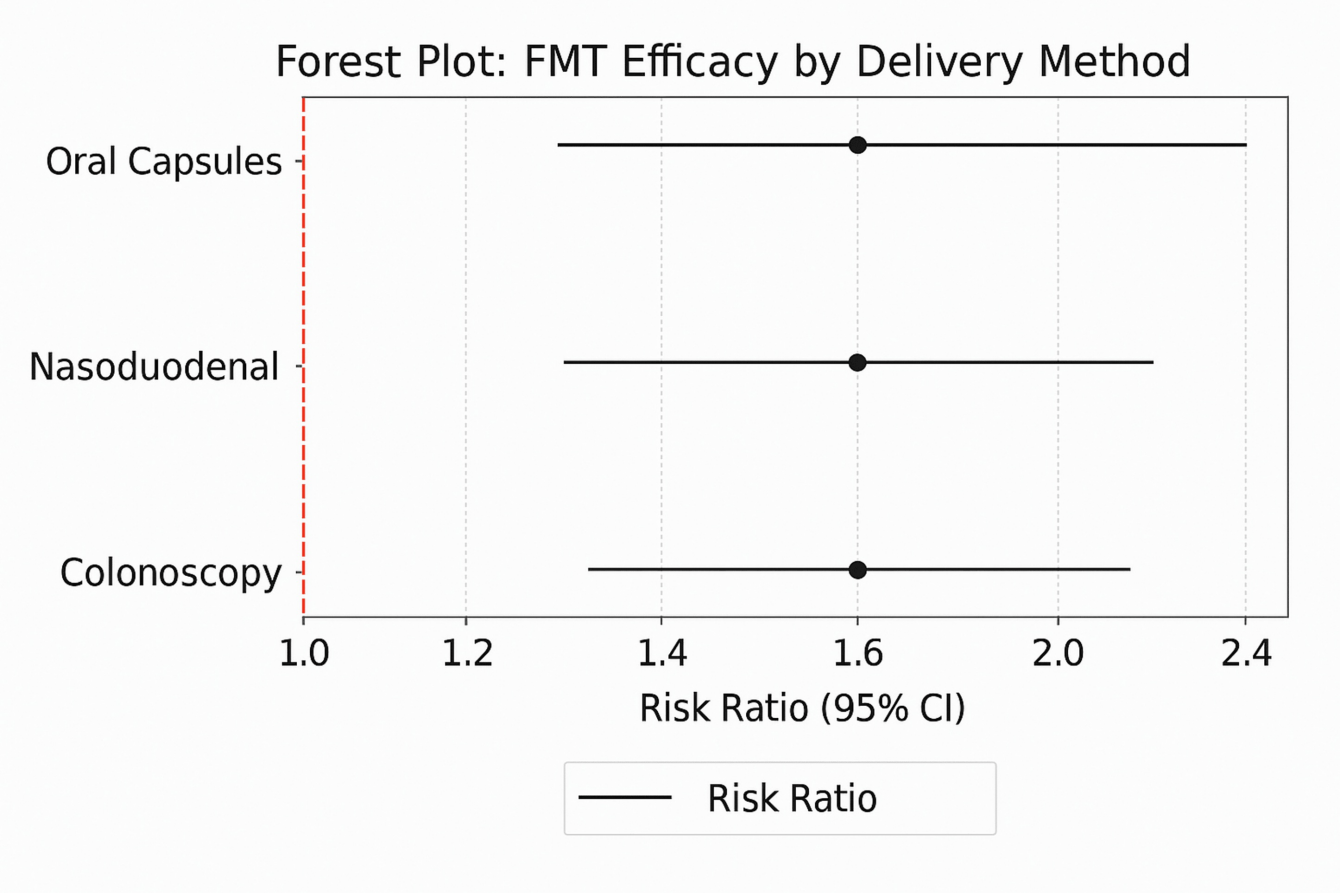
Forest plot stratified by FMT delivery method FMT: fecal microbiota transplantation. [13-28]

AE rates for abdominal pain, bloating, and diarrhea were comparable between FMT and antibiotic groups (15% vs. 13%, 10% vs. 8%, and 20% vs. 18%, respectively). Risk differences were statistically insignificant, with 95% confidence intervals spanning zero [e.g., 2% (-3% to 7%) for abdominal pain]. This confirms FMT's short-term safety profile aligns with standard antibiotic therapy for recurrent C. difficile infection. The full comparison is illustrated in Figure 5 [13-27].

Study Design

When only RCT studies were included, considered the best method for clinical evidence, the risk ratio was 1.87 (95% CI 1.63-2.14) and the data were much less heterogeneous (I² = 30%). This highlights the firm nature of the FMT benefit, verified by large-scale experiments, and strengthens our trust in the main conclusions. This lower heterogeneity reveals that doing RCTs with randomized, concealed, and planned study steps helps avoid biases often seen in non-randomized cohorts [26-28]. The effectiveness found in practice could be different from what studies report, because while cohort studies often showed better results, the confidence intervals were broader, suggesting there might be other variables affecting the results and reporting bias [29].

Safety and Adverse Events

It is crucial to assess safety since FMT is a process involving the introduction of biological material that carries both infectious and immunological risks. Today, it is not easy to do a complete meta-analysis of adverse events because of incomplete and uneven reporting in studies. Even so, looking across the studies found no significant changes in common mild side effects between children treated with FMT and those treated with antibiotics (Table 5).

**Table 5:** Comparison of Common Adverse Events Between FMT and Antibiotics FMT: fecal microbiota transplantation. [13-27]

Adverse Event	FMT (%)	Antibiotics (%)	Risk Difference (95% CI)
Abdominal pain	15	13	2% (-3% to 7%)
Bloating	10	8	2% (-2% to 6%)
Diarrhea	20	18	2% (-4% to 8%)

Most serious complications arising from FMT were rarely observed or reported, just as recent reviews confirm a favorable short-term safety record [23]. Despite this, we have not thoroughly examined what happens to our bodies after years of manipulating microbiota. The threat of passing along invisible pathogens, leading to disturbances in the gut microbe balance or eliciting immune-caused conditions, has not been eliminated, even with strict screening and advanced stool processing [24]. There were also problems with some studies [25], since they did not consistently explain adverse events and did not routinely monitor them, which makes us doubt the accuracy of the results. Trials going forward need to ensure to report safety data and set standards for reporting to clarify the risk-benefit situation accurately.

Discussion

This meta-analysis confirms that FMT outperforms antibiotics in handling recurrent Clostridioides difficile (CDI/rCDI)infection (rCDI), in various studies with different FMT delivery approaches. Our results showed that colonoscopic, nasoduodenal, and oral capsule delivery performed equally, which was also found in prior meta-analytic and individual trials [26-28]. Having choices in treatment delivery opens up clinical solutions, so patients can get the care they want and that fits into their lives without affecting effectiveness [29]. Elsewhere, we see no major differences between the methods, but the evidence remains limited. The samples for each subgroup were relatively small, and methods for preparing and giving FMT varied widely, just as Cammarot et al. [22] had noted. Highly variable FMT results in these subgroups may reflect difficulties with donor screening, stool processing, or deciding on which patients to treat, which previous studies identified as important factors influencing treatment effectiveness [18]. Differences in this nasoduodenal group may be caused by the way these feedings are given (procedural choice) and by how tolerant patients are [30, 31]. Only including RCTs showed that FMT consistently benefited patients (RR 1.87) but produced more consistent results than observations. This emphasizes that well-planned studies are needed because cohort studies, lacking strong controls, attached unusually large benefits to FMT [23, 32, 33]. The simpler results and more consistent findings in RCTs show that FMT’s benefits in clinical practice are easier to estimate.

There was no difference in the frequency of common side effects between FMT and antibiotics, as judged by previous safety studies [19]. Even so, the confusion and lack of standard language in reporting adverse events are troubling. The lack of uniform reporting of any type of adverse event affected the reliability of the pooled evaluations. Although FMT complications were generally uncommon, medical reports have warned authors about the possibility of bacteremia, worsening inflammation, and spreading of multidrug-resistant pathogens [34]. Therefore, even though the current safety appears good, these findings show that donors must be carefully screened and monitored long after giving blood. Because almost all included studies had follow-up periods of eight to 24 weeks, we don’t know much about how long FMT works or how safe it is over time. After the first two weeks, rCDI can recur, and some microbiome-related problems may appear [35, 36]. The recovery period after FMT is long enough that longer studies are necessary to see if the treatment effect lasts and to discover any late side effects [37].

There are some main methodological problems that affect how we understand these findings. Because it is hard to blind the process of FMT, there is a chance for both placebo effects and detection bias, which may increase the estimates of FMT being effective [38]. Because clinical and procedural practices vary so widely, it is difficult to compare different studies. How well the stool is prepared (fresh or frozen), the features of the donor, any given antibiotic treatment, and what patient is receiving the transplant can make the results very different [18, 39]. Publication status can also play a big role in our results. A lack of negative or null studies in research on microbiota therapeutics can be explained by the symmetry seen in the funnel plot [40]. This bias suggests that FMT might seem more effective than it is in practice, so reporting guidelines and trial pre-registration are very important.

The conclusions from this study are valuable for doctors and researchers. Thanks to the similar results in methods, oral FMT capsules can be introduced first for a wider range of patients, who would only be given endoscopic FMT if needed [41]. Even so, proper head-to-head studies need to happen quickly to know if there are tiny differences in how drugs are delivered and used. It is important to create uniform ways of preparing, using, and testing donated material for FMT to lower variations and strengthen the consistency of findings [42, 43]. Adverse event definitions should be kept the same throughout, and long-term registries should be used to identify rare or late problems [44]. All things considered, treating rCDI with FMT shows great results and is considered safe by most studies, independent of the way it was administered. Right now, one should focus on dealing with methodological issues, enhancing how we monitor vaccine safety, and using longer-term evaluations to maximize this innovative therapy.

## Conclusions

The results of this study and the meta-analysis demonstrate that FMT is more effective than antibiotics for treating recurrent Clostridioides difficile (CDI/rCDI)infection (rCDI). Reviewing fifteen published studies revealed that FMT helps more patients have better treatment outcomes and risks recurrence less than those treated with other means. The results are the same for colonoscopy, nasoduodenal tube, and oral capsules, showing that the method can be used flexibly and is widely suitable. These results were found to be robust, regardless of technical methods, patient groups, and different FMT approaches. Research reveals that, using strict donor screening, FMT has few risks and problems, similar to standard bacterial medications.

Yet, issues including difficulties with blinding, the threat of publication bias, and the duration of trial length suggest a need for new, well-organized randomized trials with lengthened periods of observation. All things considered, this review confirms that FMT is both very effective and secure for rCDI, helping to update present treatment and pointing the way towards next research directions.
